# Effects of drying temperature and relative humidity on the quality of dried onion slice

**DOI:** 10.1016/j.heliyon.2020.e04338

**Published:** 2020-07-04

**Authors:** Setia Budi Sasongko, H. Hadiyanto, Mohamad Djaeni, Arninda Mahar Perdanianti, Febiani Dwi Utari

**Affiliations:** Department of Chemical Engineering, Faculty of Engineering, Diponegoro University, Jl Prof. H. Soedarto, SH, Tembalang, Semarang, 50275, Indonesia

**Keywords:** Food science, Food technology, Degradation kinetics, Onion slice, Phenolic compounds

## Abstract

Onion, a very common season ingredient, is useful as an antioxidant and optimal conditions are required for its drying while ensuring the best quality is retained. This study evaluated the effect of drying temperatures and relative humidity on both drying rate and onion quality. Onions with an average diameter of 20.125 ± 0.025 mm were peeled and sliced into a thickness of 1.233 ± 0.029 mm. They were then dried for 120 min under various temperatures ranging from 40 to 70 °C. Both moisture content and total phenolic compounds were measured and analyzed as responses, and the data obtained were used for estimating the kinetic parameters of drying rate and total phenolic compounds degradation. The results show that the drying kinetics followed Fick's model. Moreover, the total phenolic compounds degradation can be properly expressed using a first-order reaction model, and the optimization using response surface method revealed that the optimum conditions of onion slice drying were achieved at 49.6 °C and relative humidity of 0.65%. These conditions can significantly reduce drying time with phenolic compounds retention of up to 96%.

## 1 Introduction

Onion, which is primarily used as a seasoning ingredient in several countries, is one of the most popular vegetables containing various beneficial chemical compounds such as fibers, vitamins, organic acids, phenolic compounds, and other antioxidants (Mitra et al., 2012). Phenolic compounds in onion comprise gallic acid, ferulic acid, protocatechuic acid, quercetin, and kaempferol (Cheng et al., 2013; Pérez-Gregorio et al., 2010; Singh et al., 2009), with gallic acid and quercetin being important compounds that have antiallergic, antioxidant, anti-inflammatory, antihyperglycemic, anti-lipid peroxidative, and antimicrobial properties (Balasundram et al., 2006; Cheng et al., 2013; Mitra et al., 2012). The mean daily intake of phenolic compounds is ~1756 mg (Grosso et al., 2014), which can be partially obtained by onion consumption. Phenolic compounds are highly influenced by temperature change; thus, to retain these compounds, appropriate postharvest treatments and onion storage are necessary.

Drying is one of the postharvest treatments for onion; for this process, water content is removed by introducing heat (Djaeni et al., 2014; Sharma et al., 2005). Harvested onion contains high moisture content of >80% (Asiah et al., 2017) that can be reduced to 10% or below by drying, which in turn increases storage life. Nevertheless, excessive drying can affect the stability of phenolic compounds and lead to the degradation of antioxidant compounds in onion (Kim et al., 2018). Furthermore, drying can reduce vitamin C, color, and other ingredients because of the introduction of excessive heat (Mota et al., 2010). In certain cases, in terms of energy usage, drying is inefficient (Haque and Somerville, 2013). At present, convective drying is mostly used for onion drying. To minimize energy cost, convective method via direct sun drying can be an alternative; however, it takes long drying time and is weather dependent because it requires ambient air conditions. At relatively high humidity (such as in wet season), products cannot be completely dried because of sorption isotherm characteristics.

One of the alternatives to maintain the onion quality is using an air dehumidification dryer (Djaeni et al., 2011; Zhang et al., 2007) where water content in ambient air as a drying medium is reduced by adsorptive materials such as zeolite (Djaeni et al., 2011). Note that air with a low relative humidity could enhance the drying rate at low temperature (Djaeni and van Boxtel, 2009), and therefore product quality such as nutrition and active compounds can be retained. The drying system demonstrated a good result for onion (Asiah et al., 2017) and for several other agricultural products such as wheat (Osorio-Revilla et al., 2006), roselle (Djaeni et al., 2018), and paddy (Utari et al., 2017).

Previously, studies investigated onion drying characteristics and its kinetics using different methods and under various treatments (Asiah et al., 2017; Bebartta et al., 2014; Demiray et al., 2017; Revaskar et al., 2014). Furthermore, the chemical components of onion have been studied under different process treatments (Edith et al., 2018; Sharma et al., 2015). For all cases, at higher temperature, components such as phenolic compounds can be potentially degraded. As per these facts, a comprehensive study to determine the optimum drying condition of onion is important by investigating the effect of drying temperature and relative humidity on drying time and retention of onion components such as total phenolic compounds. This study's results are beneficial for postharvest treatment of onion.

## 2 Materials and methods

### 2.1 Materials

Fresh onions of Bima variety (*Allium cepa*) having a moisture content of 83.0% (wet basis) or 4.88 kg water/kg dry solid (dry basis) were cultivated in Brebes, Central Java, Indonesia, and harvested during the wet season (April 2019). Gallic acid standard (purity 99.0% p.a., Merck, Germany), Folin–Ciocalteu reagent, Na_2_CO_3_ (2M), and ethanol (96%) were obtained from Merck, Germany, and used for analyzing total phenolic compounds in onion using a Shimadzu UV–Vis spectrophotometer (UV1800; Shimadzu Corporation, Kyoto, Japan).

### 2.2 Methods

**Figure 1:**
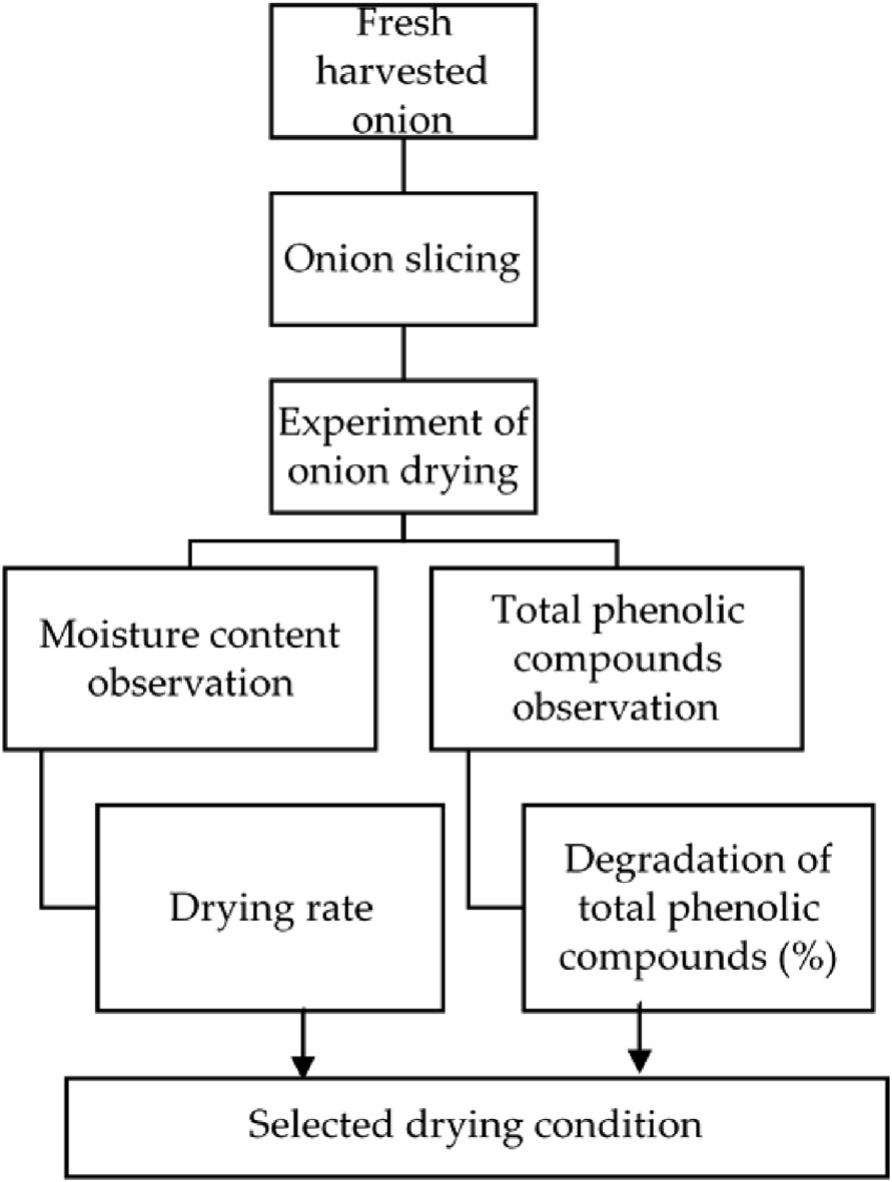
The schematic diagram of research.

Figure 1 shows the schematic of the research method that was used. Briefly, the method has four main steps: drying of onion slice under various temperatures (40, 50, 60, and 70 °C), determination of moisture and total phenolic compounds contents, kinetic model development, and selection of optimal drying conditions. Note that, using experimental data, the kinetic model was validated.

### 2.3 Onion drying

**Figure 2:**
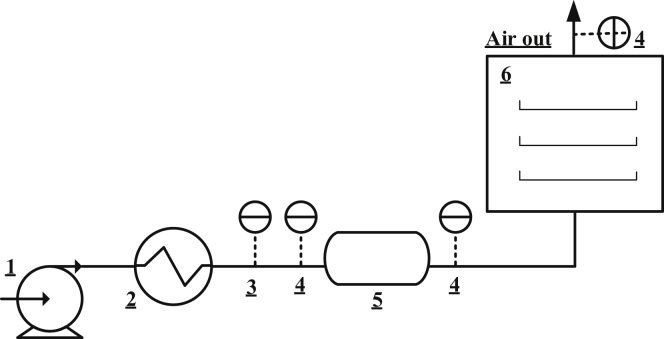
The schematic of laboratory tray dryer system: 1.Blower; 2. Heater; 3. Anemometer; 4.Temperature and relative humidity sensor (T-RH); 5. Dehumidification column and 6. Tray dryer.

Fresh onions having an average diameter of 20.125 ± 0.025 mm were peeled and sliced into a thickness of 1.233 ± 0.029 mm using a SpeedyMando slicer (Tupperware, Indonesia). They were then placed in a tray dryer using a thermocontroller. Figure 2 shows the schematic of the tray dryer. Air at room temperature of 30 °C and relative humidity of 75%–80% (measured by KW0600561, Krisbow®, Indonesia presented as T-RH) was supplied via a dehumidification column containing Zeolite 3A (provided by Zeochem) to reduce moisture content. Consequently, depending on the zeolite amount, the relative humidity of air can be kept low to a certain value (Djaeni et al., 2011). Then, air was heated to 40 °C and used for onion slice drying for 120 min (7200 s). Using an anemometer (KW0600562, Krisbow®, Indonesia), the linear velocity of air in pipe with an ID of 0.085 m was measured (~7 m s^−1^), which resulted in an air velocity of 0.54 m s^−1^ in the drying chamber (cross-sectional area = 0.15 m^2^). The moisture content in onion was measured every 10 min (600 s); however, the total phenolic compounds expressed as gallic acid equivalent were analyzed every 30 min (1800 s). These steps were then repeated for the drying temperatures of 50 °C, 60 °C, and 70 °C.

### 2.4 Kinetic model of onion drying

The kinetic model of onion drying was considered based on effective moisture diffusivity *D*_e_ (in square meters per second). This parameter can be derived using Fick's model (Ghazanfari et al., 2006). By assuming that an onion slice is a slab with a thickness *L*, Fick's model can be expressed as follows:

(1) $$MR_{,t}\;=\frac{8}{\pi^{2}}\;exp\left(\frac{-\pi 2\;D_{e}\;t}{4L^{2}}\right).$$

The moisture ratio ${\text{MR}}_{,t}$ as a function of time was calculated using Eq. (2):

(2) $$MR_{,t}=\frac{\left(M_{t}-M_{e}\right)}{\left(\;M_{0}-M_{e}\right)}$$

where $M_{t}$ is the moisture content at the time of measurement, $M_{0}$ is the initial moisture content at time 0, and *M*_e_ is the equilibrium moisture content. $M_{t}$, $M_{0}$, and *M*_e_ are expressed in kilograms of water per kilogram of dry solid. Moreover, as shown in Eq. (3), the equilibrium moisture content was calculated using the modified Henderson model (Viswanathan et al., 2003):

(3) $$1-H_{R}=exp\;(-A\left(T_{C}+C\right)\;m_{e}\;B)$$

(4) $$M_{e}=\frac{\left(m_{e}\right)}{\left(100\right)}$$

where $H_{\text{R}}$ is the relative humidity in decimal; *A*, *B*, and *C* are the equation constants with values equal to 3.6 × 10^−5^, 2.48, and 10.87, respectively (Viswanathan et al., 2003); $T_{\text{C}}\;$ is the drying temperature (in degrees Celsius); and $m_{\text{e}}$ is 100 times the equilibrium moisture content on dry basis (in kilograms of water per kilogram of dry solid).

From Eq. (3), by knowing the air relative humidity and temperature, the equilibrium moisture content in onion can be determined. Using Eq. (1), after obtaining the value of the equilibrium moisture content, the drying time (from initial moisture content of 83.0% wet basis or 4.88 kg water/kg dry solid to final moisture content of 10% wet basis or 0.11 kg water/kg dry solid) was calculated. The $D_{\text{e}}$ value was validated by moisture ratio reduction versus time from experimental data.

For expressing the relationship between effective moisture diffusivity $\left(D_{\text{e}}\right)$ and drying temperatures, the Arrhenius model (Eq. (5)) was used:_._

(5) $$ln\;D_{e}=\ln\;D_{e0}+\left(\frac{-E_{a}}{\mathrm{RT}}\right)$$

where $E_{\text{a}}$ is the activation energy of the onion drying kinetics (in kilojoules per mole), *R* is the ideal gas constant (in kilojoules per mole per Kelvin), *T* is the drying temperature (in Kelvin), and $D_{\text{e}0}\;$ is the frequency factor of the onion drying kinetics (in square meters per second).

### 2.5 Determination of total phenolic compounds in onion

Based on preliminary experiments, the extraction of total phenolic compounds was conducted. Ethanol (96%) was used as the extraction solvent because of its greater affinity to phenolic compounds compared to other solvents and because of its low toxicity (Do et al., 2014; Pohanka, 2016; Venkatesan et al., 2019). About 5 g of dried onion was mixed with 100 mL of extraction solvent. This mixture was then extracted using an ultrasound tool for 30 min and incubated in a dark room for 30 min at 30 °C, and the supernatants were used for gallic acid equivalent analysis.

Total phenolic compounds as gallic acid equivalent were analyzed using the Folin–Ciocalteu colorimetric method (Ornelas-Paz et al., 2010). The obtained extract (0.1 mL) was mixed with 50% Folin–Ciocalteu reagent (0.5 mL) and distilled water (7.9 mL). The mixture was then incubated at room temperature for 10 min. Then, sodium carbonate solution (1.5 mL; 20%) was added, and the mixture was incubated at room temperature for 60 min. The absorbance of the solution was measured using a spectrophotometer (UV1800; Shimadzu Corporation, Kyoto, Japan) at 250 nm and converted to milligrams of gallic acid equivalent per 100 g of dry onion. The calculation was then performed by calibrating the absorbance values of the sample with series of concentrations of gallic acid standards (50, 100, 200, 300, 400, 500, 600, and 700 ppm). Absorbance readings versus concentration of this series of standard solutions resulted to a linear correlation of *y* = 0.0013*x* + 0.0307 with *R*^2^ = 0.998, where *y* is the absorbance value and *x* is the gallic acid concentration (in parts per million). By knowing the sample's absorbance value, the concentration of gallic acid can be estimated, which was then converted to milligrams of gallic acid equivalent per 100 g of dry onion.

### 2.6 Fourier transform infrared spectroscopy analysis

Fourier transform infrared (FTIR) spectroscopy was used to observe the chemical composition of fresh onion and its dried product (Gierlinger, 2018). The analysis was conducted using a Frontier FT-IR 96681 spectrometer (PerkinElmer, America). FTIR spectra were recorded from 4000 to 400 cm^−1^ at a resolution of 4 cm^−1^, and sample analysis was repeated three times.

### 2.7 Kinetic model of total phenolic compounds degradation

Total phenolic compounds reported as gallic acid equivalent were analyzed every 30 min. For some food items such as apple, apple peel, and encapsulated yacon juice, total phenolic compounds degradation is expressed as a pseudo-first or first-order reaction (Arora et al., 2018; Henríquez et al., 2014; Lago and Noren, 2017), in which different process treatments and material condition and properties result in different degradation rates. Generally, the kinetics of total phenolic compounds degradation is described using a first-order reaction (Eq. (6)) as follows:

(6) $$-\frac{dC_{t}}{dt}=kC_{t}$$

where $C_{t}$ is the concentration of total phenolic compounds at sampling time (in milligrams of gallic acid equivalent per 100 g of dry onion), t is the drying time (in min), and k is the degradation rate constant of total phenolic compounds (in per min). Integration of Eq. (6) resulted in Eq. (7):

(7) $$ln\;C_{t}=ln\;C_{0}-\mathrm{kt}$$

where $C_{0}\;$ is the concentration of total phenolic compounds at initial time (in milligrams of gallic acid equivalent per 100 g of dry onion). Note that the model was fitted to the experimental data using sum of square error (SSE) and coefficient of determination (*R*^2^) for validity testing. The total phenolic compounds degradation constants can be correlated with drying temperatures using the Arrhenius correlation (Eq. (8)):

(8) $$ln\;k=ln\;k_{0}+\left(\frac{-E_{ar}}{\mathrm{RT}}\right)$$

where $E_{\text{ar}}$ and $k_{0}\;$ are the activation energy and frequency factor of the total phenolic compounds degradation kinetics expressed in kilojoules per mole and in minutes, respectively. The Arrhenius parameters $E_{\text{ar}}$ and $k_{0}\;$ were then used for predicting the value of the total phenolic compounds degradation constants at any temperature.

### 2.8 Data analysis

Statistical analysis was performed using two-way ANOVA. This method was used for testing the interaction of two independent variables such as drying time and drying temperature or drying temperature and relative humidity with a dependent variable (the total phenolic compounds). Then, the interaction of two independent variables was determined by the *p* value where *p* values ≤ 0.05 and >0.05 indicate that the effect is statistically significant and insignificant, respectively (Rydén and Alm, 2010).

### 2.9 Response surface methodology design

The drying condition was optimized based on central composite design (CCD) using Statistica 10. This experimental design comprised four cube points, four star points, and two center points. Drying temperature $\left(X_{1}\right)$ and relative humidity $\left(X_{2}\right)$ were selected as the independent variables, while total phenolic compounds degradation was selected (*Y*) as a response. Here, the second-order polynomial equation was used to correlate the factor and the response, as expressed in Eq. (9) (Mohammad et al., 2019):

(9) $$Y=\beta_{0}+\beta_{1}X_{1}+\beta_{2}X_{2}+\beta_{12}X_{1}X_{2}+\beta_{11}X_{1}^{2}+\beta_{22}X_{2}^{2}$$

where $\beta_{0}$ is the intercept constant; $\beta_{1}$ and $\beta_{2}$ are the linear effects; $\beta_{12}$ is the interaction effect; and $\beta_{11}\text{ and}$ $\beta_{22}$ are the squared effects. The 2D plot, 3D plot, and statistical analysis were generated using this software.

## 3 Results and discussion

### 3.1 Kinetic model of onion drying

#### 3.1.1 Effect of drying temperature on drying time

**Figure 3:**
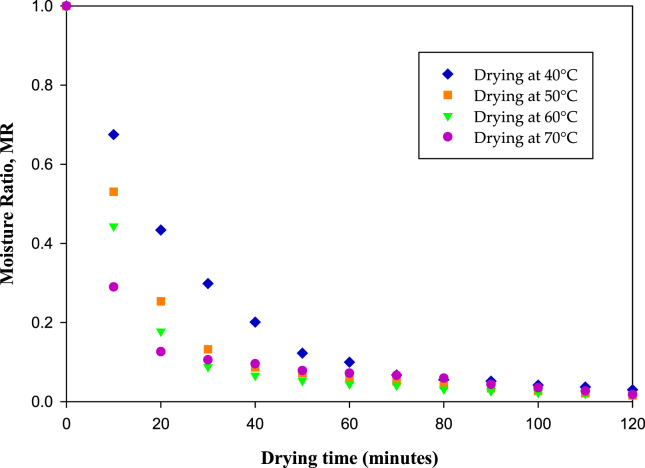
Moisture ratio versus time for drying temperatures 40, 50, 60 and 70 °C.

Moisture content during the drying process was measured every 10 min for 120 min and converted into moisture ratio, as expressed in Eq. (2). Figure 3 shows the effect of drying temperatures on moisture ratio. At the same observation time, the higher drying temperature resulted in lower moisture ratio. With increase in drying temperature from 40 °C to 50 °C, the moisture ratio was 0.5 times lesser. Compared to infrared drying, the performance of this method was higher (Pathare and Sharma, 2006). At a temperature of 40 °C and operating time of 120 min, infrared drying reduced the moisture content of fresh onion feed by ~86%, whereas the moisture content removal was 97% in this study. In this study, the drying conditions applied were superior than solar dryers working at a temperature of 30 °C and relative humidity of ~60% (Elzubeir, 2014). Solar dryers require 13 h to dry onion slice from 83% to 20% wet basis. However, in this study, at an operating temperature of 40 °C, the drying time was only 120 min (2 h).

For each drying temperature, effective moisture diffusivity was calculated using Fick's model (Eq. (1)), and the results are listed in Table 1. The effective moisture diffusivity values obtained from this research were superior compared to those of infrared drying method (Pathare and Sharma, 2006); however, they were still smaller versus those of fluidized bed working at a faster air velocity (Bebartta et al., 2014) and those of microwave onion drying with high electric power (Demiray et al., 2017). The effective diffusion coefficient value $\left(D_{\text{e}}\right)\;$ for each drying temperature can be correlated using the Arrhenius correlation as expressed in Eq. (5). Table 1 shows the parameters of the Arrhenius correlation, namely, activation energy and effective moisture diffusivity constant, were reported and validated. Using these validated parameters, $D_{\text{e}}\;$ and drying time at any operational temperature can be estimated.

**Table 1:** Kinetic parameters of drying at 40, 50, 60 and 70 °C.

T (°C)	H_R_	M_e_	$D_{e}\;$ × 10^−11^ m^2^ s^−1^	Drying time, minutes	SSE	R^2^	E_a_, kJ/mol	$D_{e0}\;$ × 10^5^ m^2^ s^−1^
40	0.400	0.097	2.219	172.967	0.046	0.986	28.549	7.323
50	0.240	0.070	3.559	87.601	0.045	0.980		
60	0.150	0.053	4.550	63.415	0.043	0.983		
70	0.080	0.039	5.917	46.170	0.067	0.965		

Drying time was estimated based on the duration of onion slice drying from initial moisture content (83.0% wet basis or 4.88 kg water/kg dry solid) to final moisture content (10% wet basis or 0.11 kg water/kg dry solid). Data from Table 1 suggests that the lower the drying temperature, the smaller is the effective moisture diffusivity; hence, moisture reduction is slower (Asiah et al., 2017; Djaeni et al., 2011, 2012).

#### 3.1.2 Effect of relative humidity on drying time

**Figure 4:**
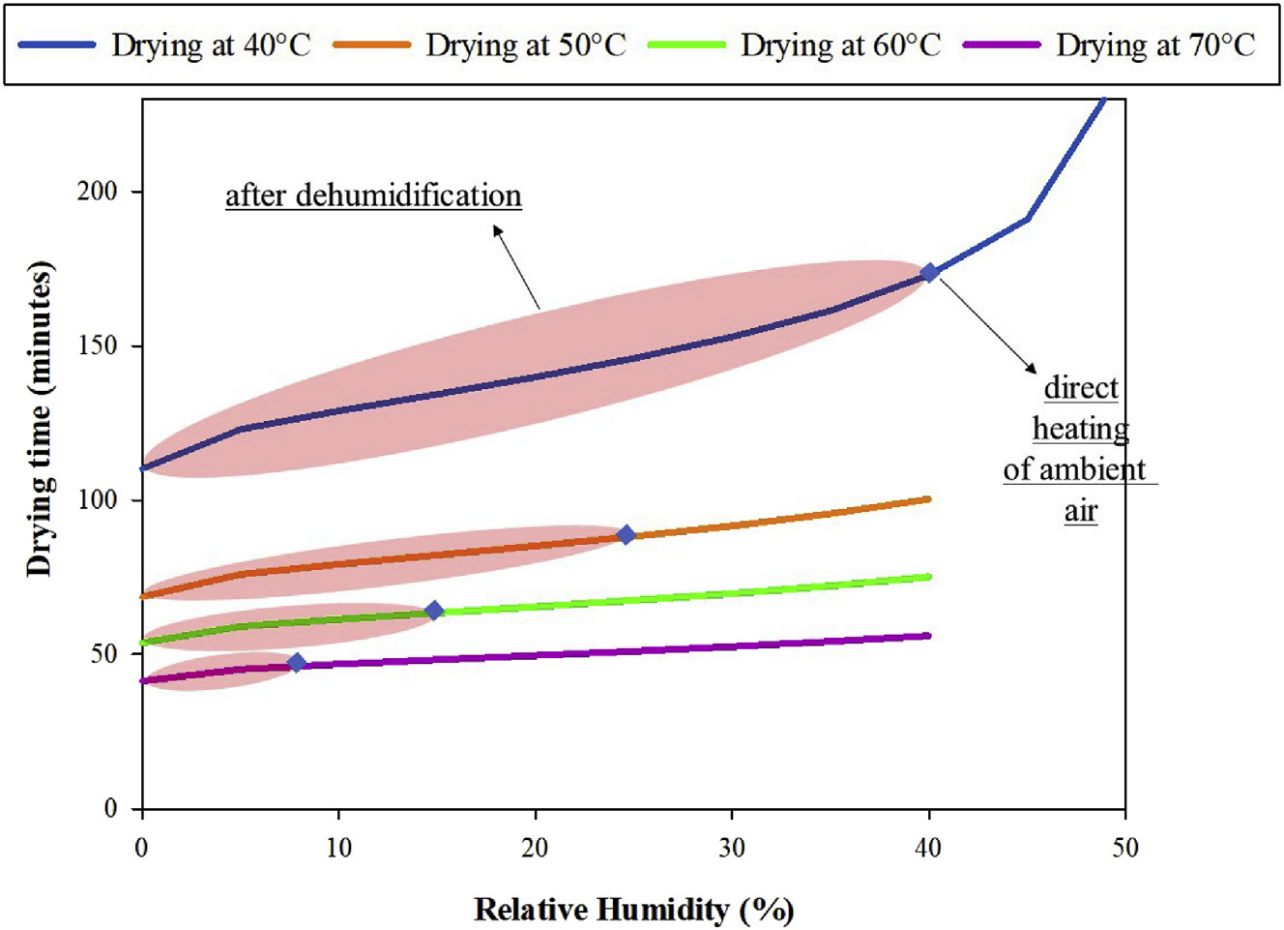
The estimated drying time at various temperatures and relative humidity.

Relative humidity influences equilibrium moisture content as expressed by the modified Henderson model (Viswanathan et al., 2003). Equilibrium moisture content is the water loaded in a material at certain temperature and relative humidity. By decreasing the relative humidity, the equilibrium moisture content in onion reduced, which enhanced moisture content reduction (Eqs. (1), (2), (3), and (4)). Relative humidity can be reduced by three possible ways: increasing operational drying temperature, removing moisture in air, and combining these two (Djaeni et al., 2011). Here, the relative humidity was varied at different drying temperatures to identify an optimum drying time; Figure 4 shows the results.

When the onion was dried with direct ambient air (temperature = 30 °C and relative humidity = 70%) as in the case of sunlight drying, the equilibrium moisture content was 0.17 kg water/kg dry solid. This result suggests that moisture content in onion cannot reach 10% wet basis or 0.11 kg water/kg dry solid, suggesting that the onion product cannot be completely dried. Note that decreasing the air relative humidity (suppose using an air dehumidification unit) can be an option to dry the onion in medium or low temperature. Using this method, the driving force of onion drying can be improved as indicated with a reduced drying time (Figure 4), e.g., in a drying temperature of 40 °C and air relative humidity of 40%, the onion can be fully dried in 172 min. With air relative humidity close to 0 and temperature at 40 °C, the drying time for onion was ~110 min. Furthermore, the effect of dehumidification was insignificant at high temperature (Djaeni et al., 2011). Drying with low relative humidity was successfully conducted in several products, such as roselle (Sasongko et al., 2019), cocoa (Abhay et al., 2016), and fruit pulp (Tiwari et al., 2012), to enhance drying driving force and retention of active or nutritional components. For all these cases, low-temperature drying can enhance product quality and reduce drying time.

### 3.2 Kinetic model of total phenolic compounds degradation

#### 3.2.1 Effect of drying temperature on total phenolic compounds

**Figure 5:**
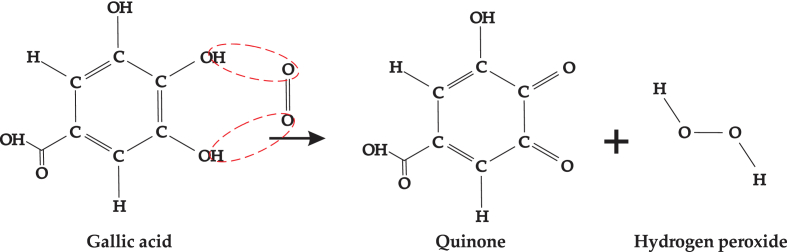
Reaction of a phenolic compound with oxygen from air forming quinone.

Phenolic compounds can be degraded by changes in lighting, pH, oxygen level, and temperature (Kim et al., 2018). In this study, the total phenolic compounds concentration expressed as gallic acid equivalent was investigated at different drying temperatures as heat treatment causes irreversible reaction of phenolic compounds. At certain pH and moisture content, phenolic compounds are trapped in the pores (Tsami and Katsioti, 2007). During drying, all parts of the thin layer of onion slice came in contact with hot air; hence, oxygen was in excess. Two atoms of oxygen from the atmosphere react with two atoms of H from the hydroxyl group to form hydrogen peroxide and quinone (Mba et al., 2019). The reaction is illustrated in Figure 5.

**Figure 6:**
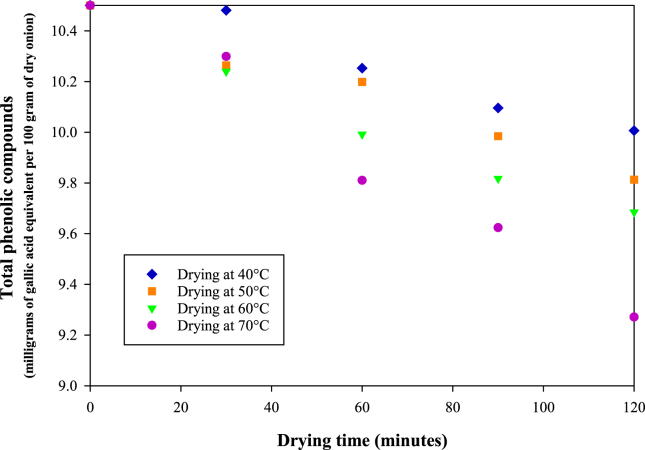
Degradation of total phenolic compounds during drying at 40, 50, 60 and 70 °C.

Figure 6 shows the effect of drying temperature on total phenolic compounds degradation. Using the two-factor test of ANOVA, the effect of drying temperature and drying time on total phenolic compounds reduction was significant because the *p* value was <0.05 (Table 2). For example, the fresh onion contained total phenolic compounds of ~10.5 mg gallic acid equivalent per 100 g of dry onion. After 120-min drying at 70 °C, ~12% of the total phenolic compounds degraded or its retention was ~88%. However, at 40 °C, the retention increased to 95% (Figure 6).

**Table 2:** Statistical result using Two-way ANOVA for the effect of drying temperature and times on the total phenolic.

Source	SS	DF	MS	F	P-value	F critical
Time	1.692	4	0.423	23.842	1.234×10^−5^	3.259
Temperature	0.365	3	0.122	6.858	0.006	3.490
Error	0.213	12	0.018			
Total	2.270	19				

SS: Sum of Squares, DF: degree of freedom, MS: Mean Squares.

**Figure 7:**
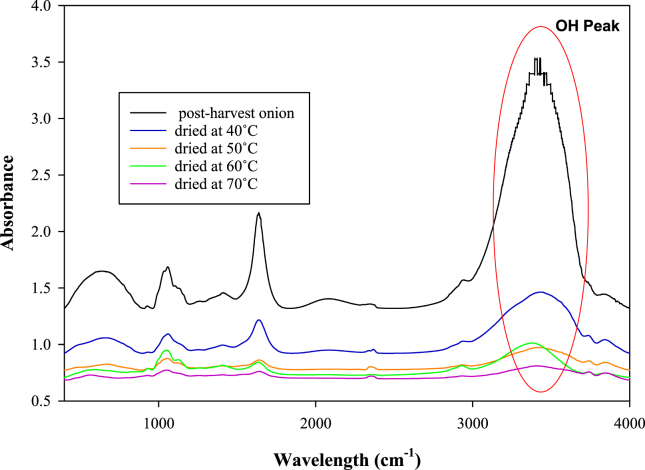
FTIR spectra of fresh and dried onion at frequency of 400–4000 cm^−1^

Phenolic compounds have a characteristic OH peak on FTIR spectra, and the absorbance of this peak is directly proportional to the concentration of phenolic compounds (Ornelas-paz et al., 2013). As shown in Figure 7, there were some absorbance changes based on FTIR analysis. After the 120-min drying process, the absorbance of the OH peak (wavelength 3,400 cm^−1^) in onion drastically decreased because of the conversion of phenolic compounds into quinone (Tulyathan et al., 1989). The amount of OH reduction increased at a higher temperature for the same drying time (120 min).

#### 3.2.2 Kinetics of total phenolic compounds degradation

The degradation of total phenolic compounds used first-order kinetic models, which were fitted to the experimental data. The parameters were evaluated by SSE and coefficient of determination (*R*^2^) with a minimum SSE value of <0.03 and maximum *R*^2^ value close to 1. Table 3 lists the value of the degradation rate constant *k* of total phenolic compounds and statistical parameters.

**Table 3:** Kinetic parameters of total phenolic compounds degradation at 40, 50, 60 and 70 °C.

T (°C)	k, minutes^−1^	SSE	R^2^	E_ar_, kJ mol^−1^	k_0_, minutes^−1^
40	4.029×10^−4^	0.012	0.953	27.043	13.065
50	5.585×10^−4^	0.006	0.980		
60	7.211×10^−4^	0.009	0.985		
70	1.017×10^−3^	0.020	0.982		

Based on the data in Table 3, as the drying temperature increased, the total phenolic compounds degradation constant increased, which indicated that at higher drying temperature, the degradation of total phenolic compounds in onion was improved. The kinetic data were then used for estimating the Arrhenius parameters, namely, activation energy $\left(E_{\text{ar}}\right)$ and $k_{0}\;$ (as expressed in Eq. (8)). The Arrhenius correlation can be used to estimate the total phenolic compounds degradation constant at any drying temperatures.

Table 3 shows the activation energy $\left(E_{\text{ar}}\right)$ and $k_{0}\;$ for the total phenolic compounds degradation in onion. In this study, the value of activation energy was 27.043 kJ mol^−1^, which is still higher than the activation energy of total phenolic compounds degradation in encapsulated yacon juice (Lago and Noren, 2017). This indicated that the degradation of total phenolic compounds in onion slice drying was lesser.

### 3.3 Evaluation on the effect of various drying conditions on total phenolic compounds

Drying kinetic parameters (Table 1) and total phenolic compounds degradation (Table 3) were considered for identifying the optimum drying time with minimum total phenolic compounds degradation (Table 4). Here, drying time is defined as the duration it will take to dry an onion slice with a moisture content of 83%–10% (wet basis). For all cases, by lowering the relative humidity, the drying time can be reduced (Figure 4) and the degradation of total phenolic compounds can be kept low (Table 4). Using the two-factor ANOVA test, the effect of drying temperature and relative humidity on the total phenolic compounds degradation was significant because the *p* value was <0.05 (Table 5).

**Table 4:** Total phenolic compounds degradation at drying temperature of 40, 50, 60, and 70 °C.

RH (%)	Drying temperature (°C)							
	40		50		60		70	
	Deg. (%)	Drying Time (min)	Deg. (%)	Drying Time (min)	Deg. (%)	Drying Time (min)	Deg. (%)	Drying Time (min)
0	4.341	110.147	3.763	68.668	3.799	53.712	4.112	41.304
5	4.834	122.978	4.155	75.983	4.167	59.022	4.485	45.134
10	5.062	128.929	4.331	79.274	4.328	61.358	4.645†	46.790†
15	5.270	134.382	4.488	82.221	4.470	63.415	4.785	48.229
20	5.482	139.944	4.645	85.151	4.608†	65.424†	4.919	49.617
25	5.712	145.997	4.809	88.239	4.751	67.498	5.055	51.028
30	5.977	152.962	4.990†	91.647†	4.904	69.729	5.199	52.520
35	6.300	161.505	5.199	95.583	5.075	72.219	5.356	54.148
40	6.731†	172.967†	5.452	100.384	5.273	75.115	5.533	55.988

† direct heating of ambient air, Deg.: Total Phenolic Compounds Degradation.

**Table 5:** Statistical result using Two-way ANOVA for the effect of drying temperature and relative humidity on the total phenolic compound degradation.

Source	SS	DF	MS	F	P-value	F critical
Relative Humidity	7.218	4	1.805	41.170	6.472×10^−7^	3.259
Temperature	9.069	3	3.023	68.967	7.770×10^−8^	3.490
Error	0.526	12	0.044			
Total	16.813	19				

SS: Sum of Squares, DF: degree of freedom, MS: Mean Squares.

With a total phenolic compounds degradation of ~4% or retention value of 96% as the basis, the optimum condition for onion slice drying was at air relative humidity of <10% with temperatures ranging from 50 to 60 °C. For comparison, in cocoa drying, decreasing the air relative humidity by zeolite significantly retained total polyphenols (Abhay et al., 2016). However, with apple dried using a drum dryer at 110–140 °C, the degradation of polyphenols was ~24.15%–45.83% (Henríquez et al., 2014). With higher polyphenol retention, onion is considered a better antioxidant because of its ability to neutralize free radicals (Cory et al., 2018; Wang et al., 2014). Owing to their anti-inflammatory property, phenolic compounds can reduce oxidative stress to prevent localized inflammation (Cory et al., 2018; Zhang and Tsao, 2016).

### 3.4 Optimization of total phenolic compounds degradation

Total phenolic compounds degradation can be inhibited at air relative humidity of <10% with temperatures ranging from 50 °C to 60 °C. Table 6 lists the conditions used for defining the low and high levels of independent variables. Table 7 lists the experimental design using orthogonal CCD comprised 10 runs.

**Table 6:** Process variables and levels of Central Composite Design (CCD) experimental design.

Independent Variables	Factor Level				
	Star Point (-1.0781)	Low Value (-1)	Center Value (0)	High Value (+1)	Star Point (+1.0781)
Temperature (°C)	49.610	50	55	60	60.390
Relative Humidity (%)	0.649	1	5.500	10	10.351

**Table 7:** The set of experimental variables using orthogonal central composite design and observed response of total phenolic compounds degradation.

Run	T (°C)	RH (%)	Response of total phenolic compounds degradation	
			Experimental value	Predicted value
1	55	6	4.391	4.451
2	60	10	4.328	4.354
3	49.610	6	4.449	4.338
4	50	10	4.331	4.399
5	60.390	6	4.337	4.305
6	50	1	3.947	4.004
7	55	0.649	4.129	4.068
8	60	1	3.973	3.988
9	55	6	4.391	4.451
10	55	10.351	4.559	4.478

As shown in Table 7, the second-order polynomial equation presented in Eq. (10) was obtained by experimental design. The model was evaluated using coefficients of determination, namely, *R*^2^ and root mean square error (RMSE) (Dailey and Vuong, 2015). The results listed in Table 8 demonstrated that both *R*^2^ and RMSE were acceptable and the model was successfully fitted.

(10) $$Y=-9.387+0.488X_{1}+0.143X_{2}-3.235\;\times 10^{-4}X_{1}X_{2}-0.004X_{1}^{2}-0.008X_{2}^{2}\;$$

**Table 8:** Regression coefficients and significance for the quadratic polynomial model of total phenolic compounds degradation.

Parameter	Regression Coefficients	Standard error	P-value
$\beta_{0}$	-9.387	0.063	2.355 ×10^−7^
$\beta_{1}$	0.488	0.079	0.718
$\beta_{2}$	0.143	0.079	0.009
$\beta_{12}$	-3.235 ×10^−4^	0.100	0.891
$\beta_{11}$	-0.004	0.121	0.141
$\beta_{22}$	-0.008	0.121	0.064
R^2^	0.892		
RMSE	0.009		

RMSE: Root Mean Square Error.

Based on the data in Table 8, relative humidity $X_{2}$ significantly influenced total phenolic compounds degradation because the *p* value was <0.05. From statistical analysis, the coefficients of drying temperature $\left(X_{1}\right)$ and relative humidity $\left(X_{2}\right)$ in Eq. (10) were positive. Note that at higher drying temperature and relative humidity, total phenolic compounds degradation increased. Furthermore, the interaction effect of drying temperature $\left(X_{1}\right)$ and relative humidity $\left(X_{2}\right)$ has a slight effect on total phenolic compounds degradation. Therefore, at a higher temperature, the effect of air relative humidity was limited.

**Figure 8:**
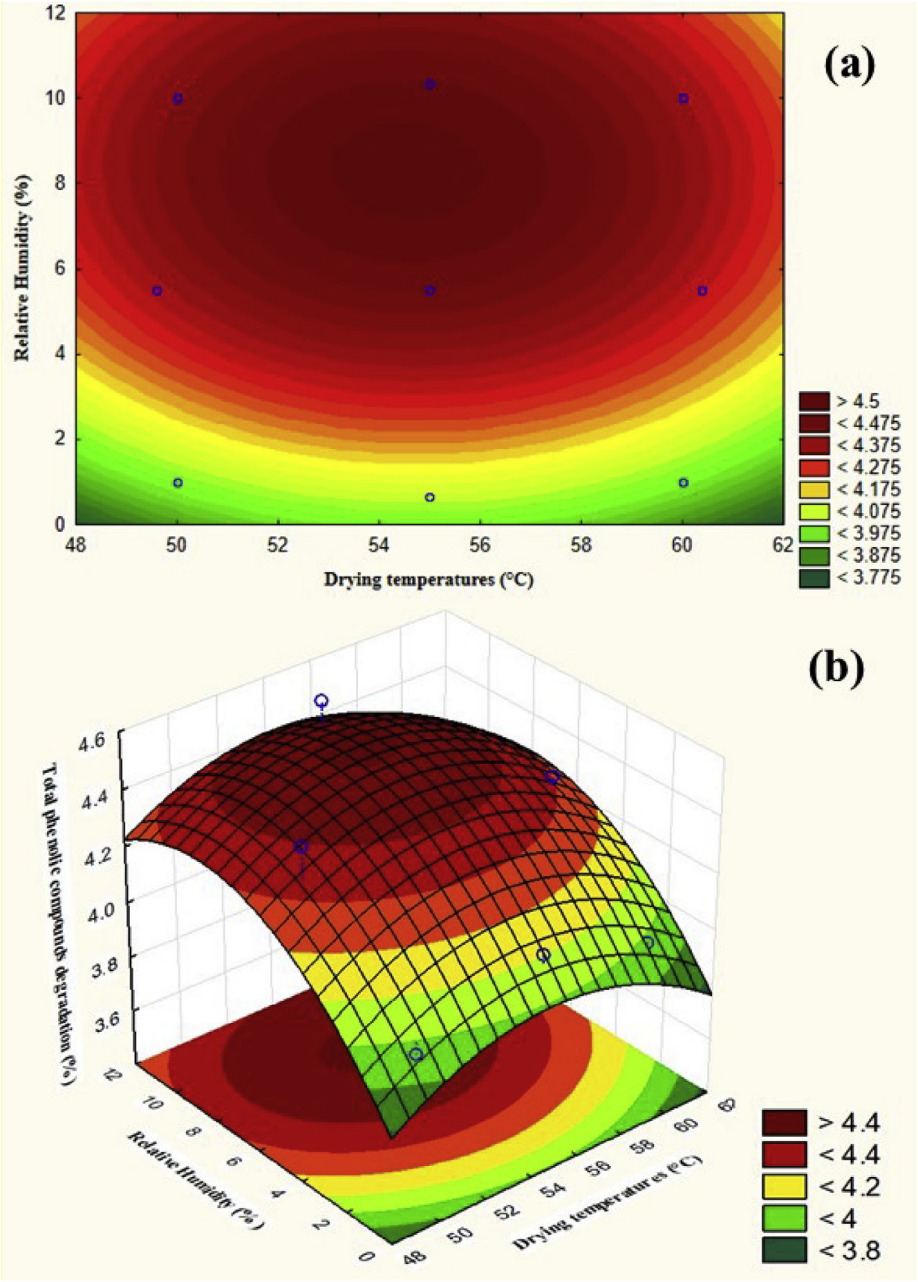
The two dimensional plot (a) and three dimensional plot (b) of total phenolic compounds degradation.

Figure 8 presents 2D and 3D plots of the total phenolic compounds degradation obtained by running Eq. (10) with the input data listed in Table 7. The result indicated that at lower drying temperature and relative humidity, total phenolic compounds degradation was inhibited. The minimum value of the degradation was 3.96%, which can be achieved at a drying temperature of 49.6 °C and relative humidity of 0.65%. In practice, decreasing relative humidity can be performed using water adsorbents, e.g., with zeolite, air can be dehumidified close to zero (Djaeni et al., 2011).

## 4 Conclusions

Phenolic compounds are important components in onion, which can be degraded by postharvest treatments using heat such as the drying process. The effects of drying conditions on moisture content and total phenolic compounds were evaluated using empirical data and model development. Phenolic compounds degradation was observed by the reduction of the OH peak on the FTIR spectra.

The results demonstrated that the drying rate of sliced onion can be expressed using Fick's model. Furthermore, the total phenolic compounds degradation can be approximately classified as a first-order reaction. Moreover, this research demonstrated that the degradation rate of polyphenol increased corresponding to the increase of drying temperature. However, at a higher temperature, the drying time for onion can be reduced. Furthermore, decreasing the relative humidity of air can enhance the driving force for drying at either low or medium temperature. Consequently, the drying time can be reduced and total phenolic compounds degradation can be minimized. Using CCD, the optimum condition for onion slice drying was achieved at a drying temperature of 49.6 °C and relative humidity of 0.65%. In these conditions, the drying time can be considerably reduced, and ~96% of the total phenolic compounds can be retained.

## Declarations

### Author contribution statement

Setia B. Sasongko: Conceived and designed the experiments; Performed the experiments; Analyzed and interpreted the data; Contributed reagents, materials, analysis tools or data.

Hadiyanto Hadiyanto, Mohamad Djaeni: Contributed reagents, materials, analysis tools or data; Wrote the paper.

Arninda M. Perdanianti: Performed the experiments; Contributed reagents, materials, analysis tools or data.

Febiani D. Utari: Analyzed and interpreted the data; Contributed reagents, materials, analysis tools or data.

### Funding statement

This work was supported by a Research for International Publication Grant provided by 10.13039/501100005844Institute of Research and Community Services, Diponegoro University (LPPM UNDIP) under contract number: 385-94/UN7.P4.3/PP/2019.

### Competing interest statement

The authors declare no conflict of interest.

### Additional information

No additional information is available for this paper.
